# The chloride/phosphate ratio combined with alkaline phosphatase as a valuable predictive marker for primary hyperparathyroidism in Chinese individuals

**DOI:** 10.1038/s41598-017-05183-6

**Published:** 2017-07-07

**Authors:** Qianqian Wang, Xu Li, Haibing Chen, Haoyong Yu, Lianxi Li, Jun Yin, Jian Zhou, Ming Li, Qing Li, Junfeng Han, Li Wei, Fang Liu, Yuqian Bao, Weiping Jia

**Affiliations:** Shanghai Diabetes Institute, Shanghai Key Laboratory of Diabetes Mellitus, Shanghai Clinical Center for Diabetes, Shanghai Jiao Tong University Affiliated Sixth People’s Hospital, Shanghai, 200233 China

## Abstract

The chloride/phosphate ratio (Cl/PO4) has been suggested to have a role in primary hyperparathyroidism (PHPT), but the associations between Cl/PO4 combined with ALP level and PHPT has not been well-studied. Our aim was to investigate the predictive value of combination Cl/PO4 with ALP for PHPT. A cross-sectional retrospective analysis was conducted to examine 172 patients diagnosed with PHPT categorized into two groups: normocalcaemic primary hyperparathyroidism (NPHPT) group and hypercalcaemia PHPT group. We found that Cl/PO4 levels and ALP levels in the NPHPT and hypercalcaemia PHPT group were both significantly higher than normal controls. Cl/PO4 and ALP levels were an independent risk factor for PHPT. Cl/PO4 combined with ALP increased the receiver-operating characteristic curves (ROC-AUC) and the diagnostic value in NPHPT and hypercalcaemia PHPT group (0.913; 95% CI, 0.744–1.000 and 0.932; 95% CI, 0.897–0.966, respectively), specificity of 92.8% and sensitivity of 98%. In conclusion, combination Cl/PO4 with ALP might be a low-cost, simple, available predictive marker of PHPT in Chinese individuals, particularly Chinese remote region where the method used to measure PTH cannot be done. Moreover, due to serum calcium level in NPHPT, Cl/PO4 combined with ALP level measurement have great potential to predict significant occurrence of NPHPT.

## Introduction

Primary hyperparathyroidism (PHPT) is characterised biochemically by an inappropriately elevated parathyroid hormone (PTH) level and it is one of the most common endocrine disorders affecting human health^1^. Using the serum calcium level, PHPT can be subdivided into normocalcaemic primary hyperparathyroidism (NPHPT) and hypercalcaemic primary hyperparathyroidism. Wills first used the term NPHPT in the 1960s^1^. NPHPT is characterised by normal serum calcium levels and consistently high PTH levels^2^. NPHPT is not a common type of PHPT. Persistently elevated PTH and hypercalcaemia are the diagnostic markers of PHPT^3^. Several studies have used the oral calcium load to diagnose PHPT inpatients with NPHPT and asymptomatic patients^4, 5^. However, in clinical, we found PTH testing did not appear in the conventional inspection indicators, and the PTH testing equipment are expensive and time-consuming. And some Chinese remote areas had no equipment measuring PTH, Simultaneously, although serum calcium was measured in most hospitals, but serum calcium level was normal in the NPHPT patients. To improve the diagnosis of PHPT, we need to find additional, simple markers for diagnosing PHPT.

Reeves introduced the concept of the chloride/phosphate ratio (Cl/PO4)^6^. Some studies found that Cl/PO4 was a good tool for distinguishing PHPT from other causes of hypercalcaemia^7–10^. Furthermore, Mismar *et al*.^11^ reported that Cl/PO4 was predictive in normocalcaemic patients in an Indian population. Katelina^12^ and McComb^9^ *et al*. suggested that alkaline phosphatase (ALP) is a membrane-bound enzyme associated with the mineralization of bone tissue. The serum ALP level is elevated in 96% of patients with primary hyperparathyroidism^13^. But there have been no study evaluating predicting diagnostic value of Cl/PO4 combined with ALP in PHPT. Therefore, this study investigated the value of the two indicators combinatiom measurement as an auxiliary diagnostic method for PHPT diagnosis in a Chinese population, especially for predicting significant occurrence of NPHPT.

## Materials and Methods

### Study Population

From January 2003 to March 2015, 172 patients with PHPT in the General Surgery Department of Shanghai Jiao Tong University Affiliated Sixth People’s Hospital were enrolled in our study. According to Chinese guidelines, for PHPT definition we used as main criteria a PTH level >65 and the presence of parathyroid adenoma based on intraoperative frozen and postoperative pathology, but not calcium blood level. PHPT can be subdivided into NPHPT and hypercalcaemic primary hyperparathyroidism using the serum calcium level. The exclusion criteria were as follows: all PHPT patients treated without surgery; secondary hyperparathyroidism; vitamin D deficiency; renal disease; hypercalciuria caused by other diseases; the use of diuretics; liver disease and other causes of an elevated PTH. The subjects were divided into two groups according to the serum calcium levels: 151 had a high serum calcium and PTH, while 19 patients were defined as NPHPT. During the same period, 50 clinical check-up healthy subjects from medical examination center of Shanghai Jiao Tong University Affiliated Sixth People’s Hospital were enrolled in our study. The inclusive criteria for them is as follows: All these participants had normal PTH levels, not suffering from PHPT disease, no family history of PHPT, normal renal function, and none were receiving medications known to influence these parameters such as sodium, potassium, ALP, phosphorus, chloride, calcium and PTH. The exclusion criteria of the control group is as follows: Patients were excluded if they had any disease that could affect electrolyte disorder (e.g. cancer, liver disease, cacotrophy, renal insufficiency, hypovolaemia and dehydration). Participants with using any related drugs such as diuretics were also excluded from the study. The study was approved by the Human Research Ethics Committee of Shanghai Sixth People’s Hospital, all experiments were performed in accordance with relevant guidelines and regulations. Each participant gave written informed consent.

### Anthropometric evaluation and biochemical measurements

General information on the 222 participants, such as gender and age, was obtained through inquiry. Anthropometric parameters such as height, body weight, and blood pressure were measured and the Body Mass Index (BMI) was calculated as weight/height2. Alanine aminotransferase (ALT) and aspartate aminotransferase (AST) were measured enzymatically on a Hitachi 7600 analyser (Hitachi, Tokyo, Japan). Serum sodium, potassium, alkaline phosphatase (ALP), phosphorus, chloride, and calcium were measured using automated techniques before surgery. ALP was detected by AMP buffer method of the ALP kit (KH-G-C-012) obtaining from Shanghai Kehua Bio-engineering Co., Ltd (Shanghai, China). The normal range for ALP is from 14 to 112 U/L. We calculated the chloride/phosphate ratio (Cl/PO4) as chloride (mEq/L)/phosphate (mg/100 mL). The total serum calcium levels used in our research was adjusted for albumin. The formula to use is: corrected calcium = measured total serum calcium in mg/dL + 0.8*(4.0- patient’s serum albumin concentration in g/dL). Serum PTH was measured by using an electrochemiluminescence immunoassay (ECLIA) on an Elecsys autoanalyser (E170; Roche Diagnostic, Mannheim, Germany). All biochemical values were measured from the same blood sample collected after an overnight fast. Related indicators were detected by Department of Medical Laboratory in Shanghai Jiao Tong University Affiliated Sixth People’s Hospital. And the detection methods of related indicators had not changed.

### Statistical analyses

Statistical analysis was performed using SPSS for Windows software (ver. 17.0; SPSS Inc., Chicago, IL, USA). Normally distributed data are presented as means ± standard deviation (SD). Non-normally distributed data determined using the Kolmogorov–Smirnov test are expressed as medians and interquartile range (25–75%). Two groups were compared using Student’s unpaired t-test. The composition ratio of categorical variables was calculated using the chi-square test. Pearson’s correlation was used to evaluate the association among the Cl/PO4 ratio, ALP and other general biochemical parameters. To analyse whether Cl/PO4 and ALP might be related to PHPT independently, we applied logistic regression models to adjust for other clinical and biochemical variables. The predictive performance of each model was assessed using area the under the receiver operating characteristic (ROC-AUC) curve with 95% confidence intervals (CI). All P-values were two-tailed and statistical significance was considered as P < 0.05.

## Results

### Subject characteristics and Serum Cl/PO4 or ALP Levels at Baseline

Table 1 summarises the clinical characteristics of the study subjects with primary hyperparathyroidism (PHPT) and normal controls. PHPT was subdivided into normocalcaemic primary hyperparathyroidism (NPHPT) and hypercalcaemia groups according to the serum calcium level. In this cross-sectional analysis of NPHPT and normal controls, no significant differences in the proportion of males, BMI, diastolic blood pressure (DBP), ALT, AST, or potassium were observed. However, patients with NPHPT were older, and had higher systolic blood pressure (SBP), ALP [107.0 (74.0–269.0) vs. 61.5 (55.8–76.3), P < 0.001], PTH, sodium, calcium [2.6 (2.5–2.6) vs. 2.4 (2.3–2.4), P = 0.001] and Cl/PO4 levels [33.9 (31.7–43.0) vs. 29.9 (27.6–32.3), P = 0.002], and a lower haemoglobin (Hb) level (P < 0.001), than controls. After adjusting for age and sex in the NPHPT group, SBP (P = 0.118) and calcium (P = 0.094) lost significance. The hypercalcaemia group of PHPT was older (P < 0.01) than the normal controls, but had a significantly (all P < 0.01) smaller proportion of males and lower BMI, Hb, and ALT. Moreover, ALP [114.0 (79.5–289.5) vs. 61.5 (55.8–76.3), P < 0.001], PTH, sodium, calcium, and Cl/PO4 [43.1 (37.0–47.5) vs. 29.9 (27.6–32.3)] were higher in the hypercalcaemic PHPT group than in the normal controls (all P < 0.001). ALT (P = 0.757) did not differ in the hypercalcaemic PHPT group after adjusting for age and sex. There were no differences in DBP, AST, or potassium levels among the groups at baseline.

**Table 1:** Baseline characteristics of the primary hyperparathyroidism (PHPT) and normal control groups.

Baseline Variables	Normal controls (n = 50)	PHPT	
		Normal calcium (n = 19)	Hypercalcaemia (n = 153)
Age (years)	45.5 ± 4.6	59.1 ± 14.3*	55.3 ± 16.0^#^
Sex male (%)	60.0	26.3	21.6^#^
BMI (kg/m^2^)	24.7 (23.3–26.9)	25.4 (23.7–25.4)	23.1 (23.1–24.1)^#‡^
SBP (mmHg)	121 (111.8–126.5)	132.0 (128.0–142.0)*	126.0 (116.0–132.0)^‡^
DBP (mmHg)	75.0 (68.8–83.3)	76.0 (70.0–80.0)	77.0 (70.0–80.0)
Hb	146.0 (135.5–158)	129.0 (124.0–141.0)*^†^	125.0 (114.0–133.5)^#‡^
ALT (U/L)	23.5 (13.8–27.5)	16.0 (13.0–21.0)	16.0 (12.0–23.0)^#^
AST (U/L)	22.5 (19.0–26.0)	19.0 (15.0–22.0)	20.0 (16.0–24.0)
ALP (U/L)	61.5 (55.8–76.3)	107.0 (74.0–269.0)*^†^	114.0 (79.5–289.5)^#‡^
Sodium (mmol/L)	140.0 (138–141.3)	142.5 (140.0–144.0)*^†^	142.0 (141.0–144.0)^#‡^
Potassium (mmol/L)	4.1 (4.0–4.3)	4.1 (3.9–4.3)	4.2 (3.9–4.4)
Calcium (mmol/L)	2.4 (2.3–2.4)	2.6 (2.5–2.6)*	2.8 (2.7–3.0)^#‡^
Phosphorus (mg/dL)	3.3 (3.0–3.6)	2.9 (2.4–3.4)*	2.6 (2.2–3.0)^#^
Chloride (mmol/L)	98.5 (97.0–100.0)	106.0 (104.0–107.0)*^†^	107.0 (105.0–109.0)^#‡^
Cl/PO_4_	29.9 (27.6–32.3)	33.9 (31.7–43.0)*^†^	43.1 (37.0–47.5)^#‡^
PTH (ng/L)	41.7 (35.0–47.8)	179.5 (131.7–435.5)*^†^	416.5 (153.2–560.6)^#‡^

Data are the means ± SD or medians (interquartile range). *P < 0.05 NPHPT subjects *vs*. normal controls; ^†^ *P* < 0.05 N*P*HPT subjects *vs*. normal controls after adjusting for age and sex; ^#^ *P* < 0.05 hypercalcaemic PHPT group *vs*. normal controls; ^‡^ *P* < 0.05 hypercalcaemic PHPT group *vs*. normal controls after adjusting for age and sex. BMI, body mass index; SBP, systolic blood pressure; DBP, diastolic blood pressure; Hb, haemoglobin; ALT, alanine aminotransferase; AST, aspartate aminotransferase; ALP, alkaline phosphatase; PTH, parathyroid hormone; Cl/PO_4_, chloride/phosphorus ratio.

### Correlation between Cl/PO4 or ALP levels and general parameters

As shown in Tables 2 and 3, correlation analysis revealed that the Cl/PO4 and ALP levels correlated with PTH and calcium in all of the subjects. Cl/PO4 levels had positive associations with age (r = 0.134, P = 0.047), ALP (r = 0.357, P < 0.001), PTH (r = 0.463, P < 0.001), sodium (r = 0.202, P = 0.003), and calcium (r = 0.586, P < 0.001) and negative associations with BMI (r = −0.251, P < 0.001) and Hb (r = −0.235, P < 0.001). Simultaneously, the ALP level was positively associated with Cl/PO4 (r = 0.357, p < 0.001), PTH (r = 0.647, p < 0.001), calcium (r = 0.58, p < 0.001), and negatively associated with BMI (r = −0.141, p = 0.036) and Hb (r = −0.307, p < 0.001). To minimise confounding effects in different groups, additional analyses were performed. Cl/PO4 and ALP levels were still significantly positively associated with PTH, calcium in both groups.

**Table 2:** Correlations of the chloride/phosphorus ratio (Cl/PO_4_) with other variables in subjects with PHPT.

Baseline Variables	Total Cohort		NPHPT		Hypercalcaemia group of PTPH	
	r	*P*-Value	r	*P*-Value	r	*P*-Value
Sex male (%)	−0.074	0.275	0.193	0.112	−0.100	0.154
Age (years)	0.134	0.047^#^	0.115	0.345	0.159	0.023^#^
BMI (kg/m^2^)	−0.251	<0.001^#^	0.076	0.532	−0.239	0.001^#^
SBP (mmHg)	0.002	0.980	0.235	0.052	0.044	0.538
DBP (mmHg)	0.109	0.106	0.209	0.084	0.113	0.109
Hb	−0.235	<0.001^#^	0.167	0.169	−0.286	<0.001^#^
ALT (U/L)	−0.094	0.164	0.050	0.683	−0.109	0.123
AST (U/L)	−0.023	0.728	−0.071	0.561	−0.033	0.644
ALP (U/L)	0.357	<0.001^#^	0.274	0.022^#^	0.419	<0.001^#^
PTH (ng/L)	0.463	<0.001^#^	0.407	0.001^#^	0.527	<0.001^#^
Sodium (mmol/L)	0.202	0.003^#^	0.251	0.037^#^	0.259	<0.001^#^
Potassium (mmol/L)	−0.056	0.408	−0.140	0.251	−0.063	0.372
Calcium (mmol/L)	0.586	<0.001^#^	0.394	0.001^#^	0.583	<0.001^#^

PHPT, primary hyperparathyroidism; NPHPT, normocalcaemic primary hyperparathyroidism; BMI, body mass index; SBP, systolic blood pressure; DBP, diastolic blood pressure; Hb, haemoglobin; ALT, alanine aminotransferase; AST, aspartate aminotransferase; ALP, alkaline phosphatase; PTH, parathyroid hormone. ^#^ *P* < 0.05 *vs*. normal controls.

**Table 3:** Correlations of ALP with other variables in subjects with PHPT.

BaselineVariables	Total Cohort		NPHPT		Hypercalcaemia group of PTPH	
	r	*P*-Value	r	*P*-Value	r	*P*-Value
Sex male (%)	−0.126	0.06	0.087	0.479	−0.142	0.043^#^
Age (year)	−0.019	0.78	0.242	0.045^#^	0.001	0.996
BMI (kg/m^2^)	−0.141	0.036^#^	0.35	0.003^#^	−0.156	0.027^#^
SBP (mmHg)	0.097	0.151	0.529	<0.001^#^	0.079	0.262
DBP (mmHg)	0.103	0.125	0.257	0.033^#^	0.123	0.08
Hb	−0.307	<0.001^#^	0.016	0.899	−0.32	<0.001^#^
ALT (U/L)	−0.029	0.663	0.169	0.165	−0.04	0.558
AST (U/L)	−0.02	0.765	0.121	0.322	−0.03	0.671
Cl/PO4	0.357	<0.001^#^	0.274	0.022^#^	0.419	<0.001^#^
PTH (ng/L)	0.647	<0.001^#^	0.623	<0.001^#^	0.648	<0.001^#^
Sodium (mmole/L)	−0.034	0.609	0.244	0.043^#^	−0.062	0.38
Potassium (mmol/L)	−0.039	0.566	0.008	0.949	−0.037	0.597
Calcium (mmol/L)	0.58	<0.001^#^	0.377	0.001^#^	0.641	<0.001^#^

PHPT, primary hyperparathyroidism; NPHPT, normocalcaemic primary hyperparathyroidism; BMI, body mass index; SBP, systolic blood pressure; DBP, diastolic blood pressure; Hb, Hemoglobin; ALT, alanine aminotransferase; AST, asparatate aminotransferase; Cl/PO4, Chloride/phosphorus ratio; PTH, parathyroid hormone; ALP, Alkalinephosphatase. ^#^p < 0.05 *vs*. normal controls.

### Baseline Cl/PO4 and ALP Level Are Predictors of PHPT

The stepwise multiple regression model was refined to assess the factors that were independently associated with PHPT (Table 4 and Table 5). Cl/PO4 levels were an independent risk factor for hypercalcaemia group of PHPT (odds ratio (OR): 0.832; 95% CI, 0.769–0.900; P < 0.001), after adjusting for age, sex, BMI, sodium and Hb. To determine if calcium had a confounding effect, the same stepwise analysis was performed in the NPHPT subgroup. Cl/PO4 levels were an independent risk factor for this group (odds ratio (OR): 0.797; 95% CI, 0.667–0.951; P = 0.012), after adjusting for some factors (Table 4). The ALP model also yielded results similar to the Cl/PO4 model (Table 5). This result shows that the Cl/PO4 and ALP levels were predictors of diagnosing PHPT.

**Table 4:** Multivariate adjusted ORs (95% CI) of Cl/PO_4_ between PHPT and normal controls.

Cl/PO_4_	NPHPT	*P*-value	Hypercalcaemia group of PHPT	*P*-value
Model 1^a^	0.878 (0.800–0.965)	0.007^#^	0.834 (0.786–0.886)	<0.001^#^
Model 2^b^	0.837 (0.735–0.952)	0.007^#^	0.832 (0.780–0.887)	<0.001^#^
Model 3^c^	0.797 (0.667–0.951)	0.012^#^	0.832 (0.769–0.900)	<0.001^#^

^a^Model 1 was unadjusted. ^b^Model 2 adjusted for age, sex, and BMI. ^c^Model 3 adjusted for the covariate in model 2 plus sodium and Hb. ^#^ *P* < 0.05.

**Table 5:** Multivariate adjusted ORs (95% CI) of ALP between PHPT and normal control.

ALP	NPHPT	*P*-value	Hypercalcaemia group of PHPT	*P*-value
Model 1^a^	0.945 (0.913–0.978)	0.001^#^	0.960 (0.944–0.976)	<0.001^#^
Model 2^b^	0.940 (0.886–0.997)	0.041^#^	0.959 (0.938–0.979)	<0.001^#^
Model 3^c^	0.929 (0.866–0.998)	0.043^#^	0.958 (0.936–0.979)	<0.001^#^

^a^Model 1 was unadjusted. ^b^Model 2 included age, sex and BMI for adjustment. ^c^Model 3 included the covariate in model 2 plus Hb for adjustment. ^#^p < 0.05.

### Predictive value of the Cl/PO4 plus ALP model

**Figure 1:**
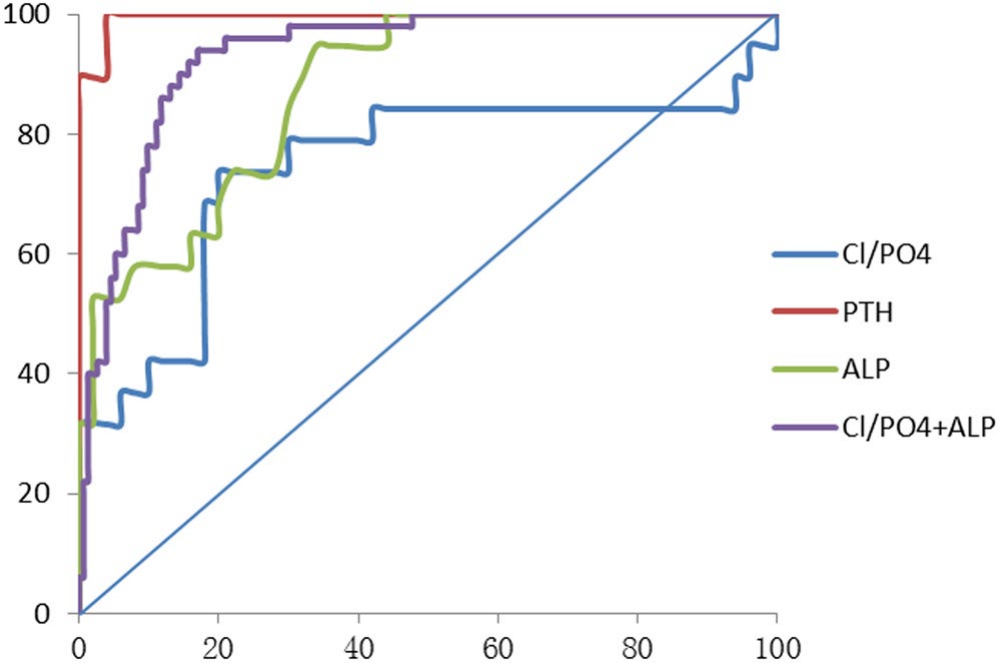
Receiver-operating characteristic curves used for predicting normocalcaemic PHPT (NPHPT) and comparison of the area under the receiver-operating characteristic curves (ROC-AUC). **Note:** Parathyroid hormone (PTH), ROC-AUC = 0.996 (95% confidence interval [CI], 0.000–1.000); chloride/phosphate ratio (Cl/PO_4_), ROC-AUC = 0.743 (95%CI, 0.587–0.899); alkaline phosphatase (ALP), ROC-AUC = 0.875 (95% CI, 0.792–0.958), Cl/PO_4_ + ALP, ROC-AUC = 0.913 (95%CI, 0.744–1.000) between normocalcaemic PHPT (NPHPT) and normal control.

**Figure 2:**
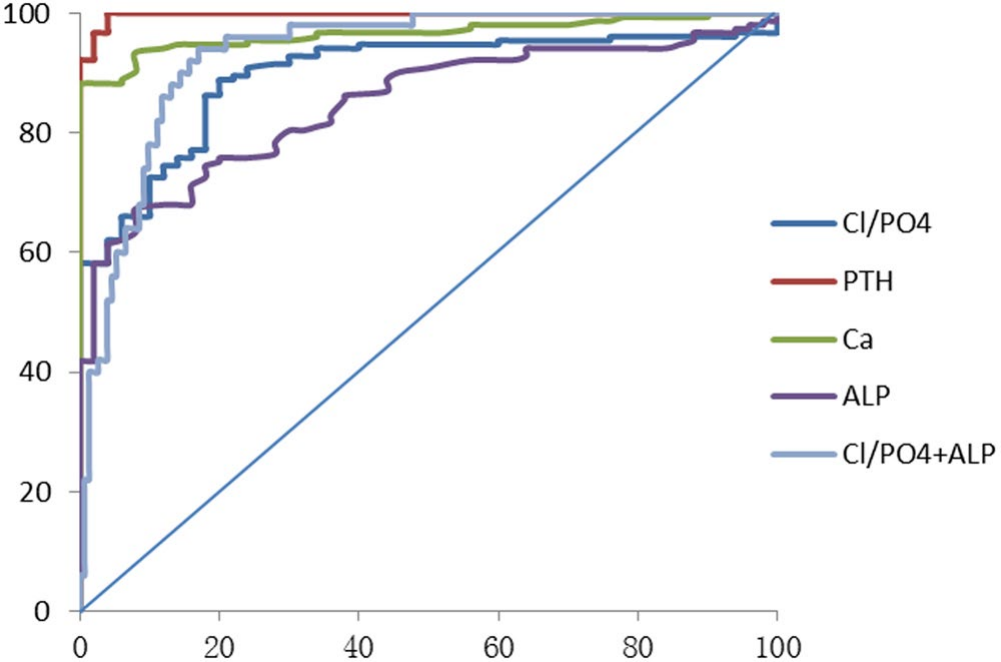
Receiver-operating characteristic curves used for predicting hypercalcaemia of PHPT and comparison of the ROC curves. **Note:** PTH, ROC-AUC = 0.998 (95% CI, 0.000–1.000), calcium, ROC-AUC = 0.966 (95% CI, 0.943–0.989), Cl/PO_4_, ROC-AUC = 0.897 (95% CI, 0.851–0.942), ALP, ROC-AUC = 0.848 (95% CI, 0.795–0.901), Cl/PO_4_ + ALP, ROC-AUC = 0.932 (95% CI, 0.897–0.966) between hypercalcaemia and normal control.

The receiver-operating characteristic curves shown in Fig. 1 represent the predictive value of different models for PHPT events in both groups. In the NPHPT group, the ROC-AUC was 0.743 (95% CI, 0.587–0.899) for Cl/PO4 alone. However, Cl/PO4 combined with the ALP model significantly increased the ROC-AUC (0.913; 95% CI, 0.744–1.000). Cl/PO4 >33.07 (specificity = 80%, sensitivity = 73.7%), ALP >71.5 (specificity = 66%, sensitivity = 94.7%), the combination of Cl/PO4 and ALP (specificity = 83%, sensitivity = 94%, positive predictive value = 53.1% and negative predictive value = 73.9%) can be used to predict diagnosing PHPT. In the hypercalcaemia group of PHPT, the ROC-AUC of Cl/PO4 alone was 0.897 (95% CI, 0.851–0.942), and increased significantly to 0.932 (95% CI, 0.897–0.966) when combined with ALP (Fig. 2). A high specificity of 80% and sensitivity of 88.9% were achieved at a Cl/PO4 threshold of 33.05. A high specificity of 92% and sensitivity of 67.3% were achieved at a ALP threshold of 90.0. When Cl/PO4 combined with ALP, specificity of 92.8%, sensitivity of 98%, positive predictive value of 51.4% and negative predictive value of 78.3% were achieved. These results show that Cl/PO4 combined with ALP might has better predictive value in both the NPHPT and hypercalcaemia groups.

## Discussion

The estimated prevalence of PHPT increased by 0.07% in 2006 in a study conducted in Scotland^14^ and by 0.09–0.23% in a study conducted in California, USA^15^. The prevalence of PHPT fluctuates between 0.27% and 0.3% with increased laboratory testing^16^. PHPT is more prevalent among postmenopausal women in China^17, 18^. The results presented here provide the first evidence that Cl/PO4 combined with ALP might increase the rate of diagnosis of PHPT in Chinese populations. This finding provides a new strategy for clinical practice. Currently, the diagnostic approach to PHPT is based mainly on serum calcium^19, 20^ and PTH^21, 22^, which has some limitations. There are no typical clinical manifestations in the early stages of PHPT, especially NPHPT, and in the diagnosis of gestational primary hyperparathyroidism (PHPT). The method used to measure intact PTH cannot be done in some hospitals, which likely causes misdiagnosis and delayed treatment. Clinicians need new screening tools to improve diagnostic accuracy.

Other auxiliary clinical indexes, such as alkaline phosphatase (ALP) levels, are increased in normocalcaemic PHPT patients^9, 23^. Boughey *et al*.^8^ analysed the data for 106 patients from South Carolina with PHPT and found that Cl/PO4 might help to confirm diagnoses of PHPT. It was useful for discriminating the cause of PHPT in addition to PTH^7, 10^. The predictive value of Cl/PO4 < 33 for excluding the diagnosis was greater than 99%. Some studies^13, 24^ have reported that serum ALP is positively associated with PTH in PHPT, and PTH significantly influenced the bone isoenzyme of ALP. Nasir *et al*.^25^ suggested ALP as a readily available marker to detect metabolic bone disorders. ALP levels were shown to be increased in PHPT patients, and serum ALP is a potential indicator for the occurrence of postoperative hypocalcemia in PHPT patients^9, 23^. Our findings which Cl/PO4 threshold >33.07 and ALP >71.5 in the NPHPT group, Cl/PO4 >33.05 and ALP threshold of 90.0 in the hypercalcaemia group of PHPT were consistent with theirs, although there were some differences due to the different populations. We found that both Cl/PO4 and ALP might be helpful biochemical predictors of PHPT. Moreover, the combination model significantly increased the predicted value of PHPT in both groups. Therefore, our finding suggests that Cl/PO4 combined with ALP is an inexpensive screening tool for predicting PHPT without the costly PTH assays.

These findings provide important clues regarding the pathophysiological mechanism underlying the association between Cl/PO4 and PHPT. One possible explanation is that PTH decreases the tubular reabsorption of phosphate in the proximal renal tubule, which increases urinary phosphate and decreases serum phosphate^25, 26^. The reabsorption of bicarbonate from the proximal renal tubule is decreased by the action of PTH, causing the reabsorption of chloride and increasing the secretion of bicarbonate from the kidney^27^. These findings may partly account for the observed association between serum Cl/PO4 and PHPT.

Previous studies^25, 28–30^ suggested that elevated ALP levels existed in the patients of primary hyperparathyroidism. Serum alkaline phosphatase originated principally from bone. Elevated level of ALP is a marker of osteoblast activity increase. Bone metabolism is a dynamic balance of the process by common regulation of bone resorption and bone formation. Although osteoclasts are the primary target organ for PTH to accelerate bone resorption and destruction, functional PTH receptors were expressed in both osteoblasts and osteoclasts. When PTH combined with its receptors, they could indirectly stimulate effect mediated by osteoblasts. Due to bone formation and bone resorption processes coupling in bone remodeling units, osteoblast activity also increased in PHPT with the increase in osteoclast activity. Osteoblasts responded to PTH with an increase in ALP as bone morphogenetic marker. Additionally, as the research was observing this trend of ALP slight elevation currently, the further studies including more samples should be performed. So in clinical work, when we found that ALP was close to upper limit of the normal range or higher than normal range, we need to further detect other laboratory indicators of PHPT so that they could increase the predicted value of PHPT.

The above findings may partly account for the observed association among serum Cl/PO4, ALP and PHPT. Simultaneously, in the study, we found significant difference of Hb and sodium between patients with hyperparathyroidism and normal, but there was no significant difference after adjusting for Hb and sodium. On the one hand, the pathophysiological mechanism of elevated Hb in PHPT patients has not yet known. On the other hand, as for serum sodium level was different in two groups, one possible pathophysiological mechanism was that patients with hyperparathyroidism have higher aldosterone levels, and elevated aldosterone levels have positive correlation with sodium intake^30^. Another possible pathophysiological mechanism was that the reabsorption of sodium was induced by PTH^25, 26^. Further research is needed to clarify the potential pathophysiological mechanism. This study had several limitations. First, the age and sex of control group were not very match up to the PHPT group, so we performed the analysis adjusting for age and sex in each step to eliminate the bias from age and sex. Second, a small sample size from a single centre cannot fully reflect the entire Chinese population. The study was based on parathyroid adenoma of PHPT. Further prospective studies that include parathyroid carcinoma and parathyroid hyperplasia cases should be performed. Additionally, we have analysed the patients of PTH-dependent hypercalcemia in the study. Whether the chloride/phosphate ratio combined with alkaline phosphatase as a valuable predictive marker for PTH-independent hypercalcemia, further study is urged to for certification. Third, given the cross-sectional design of the study, the most important limitation is a lack of causation. Therefore, follow-up studies should be prospective and include a large number of cases to confirm our findings.

In conclusion, we revealed an association among Cl/PO4, ALP and PHPT, especially NPHPT, in Chinese individuals. Due to its low cost and availability, Cl/PO4 could increase the rate of diagnosis of PHPT, particularly NPHPT, when combined with ALP. The clinical significance of this model should be corroborated in larger studies.
